# Non-invasive Monitoring of Intracranial Pressure Using Transcranial Doppler Ultrasonography: Is It Possible?

**DOI:** 10.1007/s12028-016-0258-6

**Published:** 2016-03-03

**Authors:** Danilo Cardim, C. Robba, M. Bohdanowicz, J. Donnelly, B. Cabella, X. Liu, M. Cabeleira, P. Smielewski, B. Schmidt, M. Czosnyka

**Affiliations:** 1 Brain Physics Laboratory, Division of Neurosurgery, Department of Clinical Neurosciences, University of Cambridge, Box 167, Cambridge Biomedical Campus, Cambridge, CB2 0QQ UK; 2 Neurosciences Critical Care Unit, Addenbrooke’s Hospital, Cambridge University Hospitals NHS Foundation, Cambridge, UK; 3 Institute of Electronic Systems, Warsaw University of Technology, Warsaw, Poland; 4 Department of Neurology, University Hospital Chemnitz, Chemnitz, Germany

**Keywords:** Non-invasive intracranial pressure monitoring, Transcranial Doppler Ultrasonography, Intracranial pressure

## Abstract

Although intracranial pressure (ICP) is essential to guide management of patients suffering from acute brain diseases, this signal is often neglected outside the neurocritical care environment. This is mainly attributed to the intrinsic risks of the available invasive techniques, which have prevented ICP monitoring in many conditions affecting the intracranial homeostasis, from mild traumatic brain injury to liver encephalopathy. In such scenario, methods for non-invasive monitoring of ICP (nICP) could improve clinical management of these conditions. A review of the literature was performed on PUBMED using the search keywords ‘Transcranial Doppler non-invasive intracranial pressure.’ Transcranial Doppler (TCD) is a technique primarily aimed at assessing the cerebrovascular dynamics through the cerebral blood flow velocity (FV). Its applicability for nICP assessment emerged from observation that some TCD-derived parameters change during increase of ICP, such as the shape of FV pulse waveform or pulsatility index. Methods were grouped as: based on TCD pulsatility index; aimed at non-invasive estimation of cerebral perfusion pressure and model-based methods. Published studies present with different accuracies, with prediction abilities (AUCs) for detection of ICP ≥20 mmHg ranging from 0.62 to 0.92. This discrepancy could result from inconsistent assessment measures and application in different conditions, from traumatic brain injury to hydrocephalus and stroke. Most of the reports stress a potential advantage of TCD as it provides the possibility to monitor changes of ICP in time. Overall accuracy for TCD-based methods ranges around ±12 mmHg, with a great potential of tracing dynamical changes of ICP in time, particularly those of vasogenic nature.

## Introduction

Intracranial pressure (ICP) is an important monitoring modality in the clinical management of several neurological diseases carrying intrinsic risk of potentially lethal intracranial hypertension (ICH). ICP essentially consists of four components, driven by different physiological mechanisms [1]: inflow and volume of arterial blood, venous blood outflow, cerebrospinal fluid (CSF) circulation, and brain volume. These components are responsible for different patterns of ICH.

Although ICP can guide patient management in neurocritical care, it is not commonly monitored in many clinical conditions outside this environment. The invasive character of the standard methods for ICP monitoring (epidural, subdural, intraparenchymal, and intraventricular monitors) and their associated risks to the patient (infections, brain tissue lesions, and hemorrhage) contribute to this scenario. They have prevented ICP monitoring in a broad range of diseases like in patients with risk of coagulopathy, as well as in other conditions in which invasive monitoring is not considered or outweighed by the risks of the procedure. Another downside is related to costs and availability: invasive monitoring is an expensive technique, requires trained personnel and neurosurgical settings. Average cost of intraparenchymal microtransducer is US $600, additionally to US $6000–10,000 for the display monitor [2]. Provided that knowledge of ICP can be crucial for the successful management of patients in many sub-critical conditions, non-invasive estimation of ICP (nICP) may be helpful when indications for invasive ICP monitoring are not met and when it is not immediately available or even contraindicated.

Transcranial Doppler Ultrasonography (TCD) was first described by Aaslid et al. [3]. Apart from many clinical applications, TCD waveform analysis has been investigated as a technique for nICP estimation, and this could represent one of its most useful applications outside the critical care environment. It is conceivable if one considers that increased ICP could affect the waveform of blood flow velocity in major cerebral vessels which have compliant walls. Such vessels are subjected to an external pressure (ICP) and an internal pressure (arterial blood pressure—ABP). Active tension of the arterial walls and the arterial wall compliance are another (and unknown) parameters, which undoubtedly fall into the equation. On top of this, not only all changes in FV waveform, like low diastolic cerebral blood flow velocity (FVd), peaked waveform, and higher pulsatility index (PI) values can be observed with TCD during elevated ICP, but also in arterial hypotension and hypocapnia [4, 5].

TCD-based nICP methods are mainly based on approximate semi-quantitative relationships between cerebrovascular dynamics and ICP. They can be divided into three categories: (I) methods based on the TCD-derived pulsatility index; (II) methods based on the calculation of non-invasive cerebral perfusion pressure (nCPP); and (III) methods based on mathematical models.

Although derived from the same principle, there is a considerable variability in the reported accuracy of these methods inter- and intra-categories.

Considering the wide range of TCD applications as a technique for nICP monitoring, the purpose of this review is to generally present these methods and their documented clinical or experimental applications with measures of accuracy.

## Methods

**Fig. 1:**
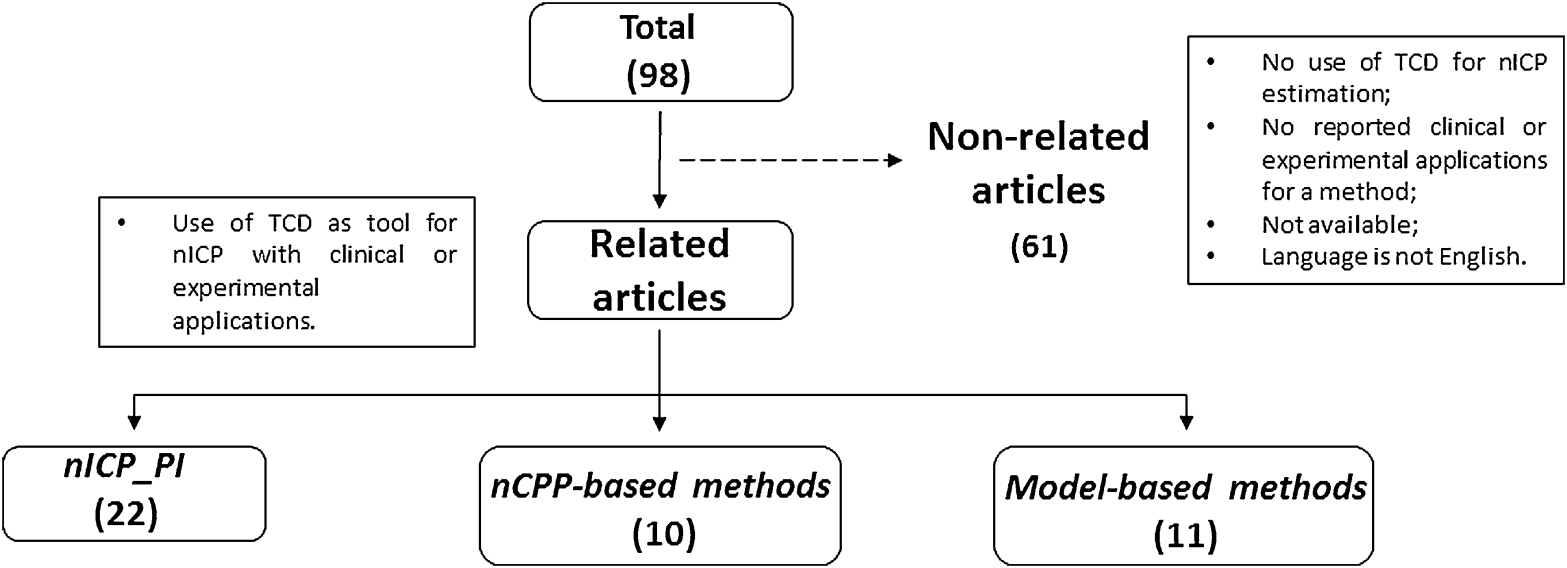
Flow diagram representative of the methodological approach applied for the selection of articles

A review of the literature was performed on PUBMED database using the search keywords ‘Transcranial Doppler non-invasive intracranial pressure.’ Works from 1985 to 2015 were found, in a total of 98 studies. The inclusion criteria were the use of Transcranial Doppler as tool for non-invasive ICP with clinical or experimental applications of such methods. Excluded papers consisted of works using TCD, but with no application for non-invasive ICP estimation, or absence of clinical or experimental applications in papers describing methods using TCD for non-invasive ICP estimation. The selected articles were then subdivided into the three categories aforementioned. Only full-length available articles in the English language were considered (see flow diagram in Fig. 1). The total number of articles considered was 37, with occasional repetitions within each nICP category, i.e., certain articles presented assessments in more than one category.

Results for each nICP category are presented in Tables 1, 2, and 3. They include main findings and measures of accuracy of the studies considered. Sections in Results present major attributes of the nICP categories.

**Table 1:** nICP methods based on TCD-derived Pulsatility Index (*nICP_PI*)

Method	Author	Study purpose	Sample size and disease	Invasive ICP monitoring	nICP method accuracy and correlation measures with ICP	Sensitivity (%)	Specificity (%)	AUC	PPV (%)	NPV (%)
*nICP_PI*	Steiger [52]	Investigate PI in TBI patients and compare them to healthy volunteers	9, TBI	NA	PI analysis revealed values from 1.5 to 2.0 in control subjects, showing a gradual increase in patients with post-traumatic brain oedema. PI values ≥3 were associated with severe intracranial hypertension					
	Chan et al. [53]	Examine the relationships between FV, SJO_2_, and alterations in ABP, ICP, and CPP	41, TBI	Subdural	Rises in ICP or drops in ABP were associated with a reduction in FV, particularly with FVd falling more than FVs. PI was strongly correlated with ICP (*R* = 0.9*)					
	Homburg et al. [54]	Investigate the PI-ICP relation as to evaluate TCD as an alternative to invasive ICP	10, TBI	Epidural	Correlation between PI and ICP was *R* = 0.82					
	Martin et al. [55]	Assess PI in three distinct haemodynamic phases (hypoperfusion, hyperaemia and vasospasm)	125, TBI	Intraventricular/Intraparenchymal	Higher PI values were found in all hemodynamic phases during the first 2 weeks after injury (on day 0, compared to days 1–3 and days 4 through 14 post-trauma)					
	McQuire et al. [56]	Investigate the incidence of early abnormalities in the cerebral circulation after TBI by relating the results of CT scan with TCD-PI	22, TBI	NM	Ten patients presented increased PI in conditions indicative of ICH (space-occupying hematomas or brain swelling indications on CT)					
	Moreno et al. [57]	Investigate the correlation between TCD, ICP and CPP in TBI patients	125, TBI	NA	Correlation between PI and ICP was *R* ^2^ = 0.69. Elevated PI (≥1.56) was predictive of poor outcome					
	Rainov et al. [51]	Investigate a possible relationship between PI, RI, FV and ICP changes in adult patients with hydrocephalus	29, Hydrocephalus	Epidural	PI in patients with elevated ICP prior to shunting was significantly increased. Preshunting ICP and PI were not correlated (*R* = 0.37)					
	Asil et al. [58]	TCD was compared with clinical examination and neuroradiologic findings	18, Stroke and MCA infarction	NM	Increases in PI were correlated with midline shift as indication of elevated ICP (*R* = 0.66*)					
	Bellner et al. [7]	Investigate the relationship between ICP and PI in neurosurgical patients	81, (SAH, TBI and other intracranial disorders)	Intraventricular	Correlation between PI and ICP was *R* = 0.94* (ICP = 10.93 × PI − 1.28) In the ICP range of 5–40 mmHg, the correlation formula is: ICP = 11.5 × PI − 2.23 (*R* ^2^ = 0.73*) In this interval, SD for *nICP_PI* was ±2.5 mmHg in the ICP range of 5-40 mmHg 95 % CI of ±4.2 mmHg	88 (threshold of 10 mmHg) 83 (threshold of 20 mmHg)	69 (threshold of 10 mmHg) 99 (threshold of 20 mmHg)			
	Voulgaris et al. [59]	Investigate TCD as tool for detection of cerebral haemodynamics changes.	37, TBI	Intraparenchymal	Overall correlation between ICP and PI was *R* = 0.64* For ICP ≥ 20 mmHg, correlation was *R* = 0.82* PI allows early identification of patients with low CPP and risk of cerebral ischemia					
	Behrens et al. [49]	Validate TCD as a method for ICP determination INPH	10, INPH	Intraparenchymal	Correlation between PI and ICP was *R* ^2^ = 0.22* (ICP = 23 × PI + 14) 95 % CI for a mean ICP of 20 mmHg was −3.8 to 43.8 mmHg PI is not a reliable predictor of ICP					
	Figaji et al. [47]	Examine the relationship between PI and ICP and CPP in children with severe TBI	34 children, TBI	NM	Marginal correlation between PI and ICP of *R* = 0.36* No significant relationships between PI and ICP when differences within individuals or binary examination of PI (PI < 1 and ≥1) were considered PI is not a reliable non-invasive indicator of ICP in children with severe TBI	25 (threshold of 20 mmHg)	88 (threshold of 20 mmHg)			
	Brandi et al. [60]	Assess an optimal nICP and nCPP following TBI using TCD	45, TBI	Intraventricular	Bellner’s equation resulted in nICP similar to measured ICP, with nICP of 10.6 ± 4.8 and ICP of 10.3 ± 2.8 mmHg					
	Tude Melo et al. [61]	Evaluate the accuracy of TCD in emergency settings to predict intracranial hypertension and abnormal CPP in children with TBI	117 children, TBI	Intraparenchymal	PI ≥ 1.31 was observed in 94 % of cases with initially elevated ICP, and 59 % of those with normal initial ICP values TCD is an excellent first-line examination to screen children who need urgent treatment and continuous invasive ICP monitoring	94 (for detecting initial ICH)	95 (for detecting initial ICH)			
	Zweifel et al. [48]	Assess PI as a diagnostic tool for nICP and nCPP estimation	290, TBI	Intraparenchymal	Correlation between PI and ICP was *R* = 0.31* 95 % prediction interval > ±15 mmHg The value of PI to assess nICP is very limited			0.62 (ICP ≥ 15 mmHg) 0.74 (ICP ≥ 35 mmHg)		
	De Riva et al. [6]	Assess the relationship between PI and CVR in situations where CVR increases (mild hypocapnia) and decreases (plateau waves of ICP) in TBI patients	345, TBI	Intraparenchymal	Correlation between PI and ICP in such situations was *R* = 0.70* 95 % CI of ± 21 mmHg					
	Wakerley et al. [62]	Assess the correlation between PI with CSF pressure	78, miscellaneous intracranial disorders	LP	Correlation between PI and ICP (CSF pressure) was *R* = 0.65* Binomial logistic regression indicated a strong significant relationship between raised ICP and PI (OR 2.44; 95 % CI 1.57–3.78)	81.1	96.3	0.84 (threshold ≥20 cmH_2_0)		
	Wakerley et al. [63]	Present a case where TCD serves as an effective tool for nICP monitoring	Case-report, Sagittal Sinus Thrombosis	NM	Increasing ICP was associated with rapid elevations of PI. On recording day 4, PI was reported to be 1.93 (considering a normal range of 0.6-1.2). ICP was deemed elevated according to clinical status (level of consciousness, headache) and papilledema					
	O’Brien et al. [64]	Determine the relationship between PI, FV_d_ and ICP in children with severe TBI.	36 children, TBI	Intraventricular/Intraparenchymal	Initial 24 h post-injury: Correlation between PI and ICP was *R* = 0.6* Beyond 24 h post-injury: Correlation between PI and ICP was *R* = 0.38*	Initial 24 h post-injury: 100 (threshold ≥ 20 mmHg for PI of 1.3) Beyond 24 h post-injury: 47 (threshold ≥ 20 mmHg for PI of 1.3)	Initial 24 h post-injury: 82 (threshold ≥ 20 mmHg for PI of 1.3)		88.1	90.1
	Robba et al. [65]	Assess PI as a nICP estimator in rabbits submitted to infusion of artificial CSF solution into the subarachnoid space.	Experimental (n = 28 rabbits)	Intraparenchymal	Correlation between PI and ICP was *R* = 0.54* *R* considering changes of nICP and ICP in time domain was 0.36 ± 0.47 95 % CI of ± 38.56 mmHg.			0.62 (≥20 mmHg) 0.66 (≥40 mmHg)		
	Cardim et al. [19]	Assess PI as a nICP estimator in TBI patients and compares it with 3 other methods.	40, TBI	Intraparenchymal	Correlation between PI and ICP was *R* = 0.15 Bias of 4.11 ± 4.90 and 95 % CI of ±9.62 mmHg *R* considering changes of nICP and ICP in time domain was 0.61 ± 0.35.			0.70 (≥17 mmHg)		
	Cardim et al. [44]	Assess PI as a nICP estimator and compares it with 3 other methods in NPH patients undertaking CSF infusion tests	53, NPH	Lumbar puncture (CSF pressure)	Baseline phase of the test: Bias of 8.91 mmHg and 95 % CI of ±10.58 mmHg Plateau phase of the test: Bias of −6.18 mmHg and 95 % CI of ±19.07 mmHg ΔICP vs ΔnICP (magnitude of increase in ICP) correlation of 0.45* *R* considering changes of nICP and ICP in time domain was 0.39 ± 0.40					

* Correlation coefficient is significant at the 0.05 level

ABP, arterial blood pressure, *AUC*, area under the curve;* CI* confidence interval; *CT*, computerized tomography;* CSF* cerebrospinal fluid;* FVd* diastolic flow velocity;* ICH* intracranial hypertension;* INPH* idiopathic normal pressure hydrocephalus;* LP* lumbar puncture;* MCA* middle cerebral artery;* NA* not available;* NM* not measured;* NPV* negative predictive value;* OR* odds ratio;* PPV* positive predictive value;* R* correlation coefficient;* R* ^2^ coefficient of determination; ;* RI* resistance index;* SAH* subarachnoid haemorrhage;* SD* standard deviation;* SJO*_2_ jugular bulb venous blood oxygen saturation;* TBI* traumatic brain injury

**Table 2:** nICP methods based on the non-invasive cerebral perfusion pressure

Method	Author	Study purpose	Sample size and disease	Invasive ICP monitoring	nICP/nCPP method accuracy and correlation measures with ICP/CPP	AUC	PPV (%)	NPV (%)
*nICP_Aaslid*	Aaslid et al. [8]	Describe and assess a method for nCPP calculation based on FV and ABP	10, supratentorial hydrocephalus	Intraventricular	SD for nCPP estimation of 8.2 mmHg at 40 mmHg Able to differentiate between low (≤40 mmHg) and normal (≥80 mmHg) CPP in all cases Low accuracy (80 %) at higher levels of CPP (70–100 mmHg)			
	Czosnyka et al. [10]	Assessment of *nICP_Aaslid*	96, TBI	Intraparenchymal	95 % PE for nCPP estimation was >27 mmHg Sensitive to detect changes of CPP over time			
	Robba et al. [65]	Assessment of *nICP_Aaslid*	Experimental (n = 28 rabbits)	Intraparenchymal	Correlation between nICP and ICP was *R* = 0.53* *R* in time domain of 0.61 ± 0.35 95 % CI of ±59.60 mmHg.	0.66 (≥20 mmHg) 0.77 (≥40 mmHg)		
*nICP_FVd*	Czosnyka et al. [10]	Describe and assess a method for nCPP calculation based on FV (using the concept of FVd) and ABP	96, TBI	Intraparenchymal	Correlation between nCPP and CPP was *R* = 0.73* Estimation error was less than 10 and 15 mmHg in 71 and 84 % of the cases, respectively Highly specific for detecting changes over time: *R* ^2^ = 0.82 Averaged correlation considering day-by-day variability between CPP and nCPP was *R* = 0.71		94 (CPP ≤ 60 mmHg)	
	Schmidt et al. [66]	Assessment of *nICP_FVd*	25, TBI	Intraparenchymal	Error for nCPP estimation was less than 10 and 13 mmHg in 89 and 92 % of the cases respectively 95 % CI of ±12 mmHg			
	Gura et al. [67]	Assessment of *nICP_FVd*	47, TBI	Intraparenchymal	Correlation between nCPP and CPP was *R* = 0.92* Mean values of nCPP and CPP were 66.10 ± 10.55 mmHg and 65.40 ± 10.03 mmHg, respectively			
	Brandi et al. [60]	Assess an optimal nICP and nCPP following TBI using TCD	45, TBI	Intraparenchymal	nICP: Bias of 5.6 mmHg and 95 % CI of ± 17.4 mmHg nCPP: Bias of −5.5 mmHg and 95 % CI of ± 20.6 mmHg.			
	Robba et al. [65]	Assessment of *nICP_FVd*	Experimental (n = 28)	Intraparenchymal	Correlation between ICP and nICP was *R* = 0.77* *R* in time domain of 0.85 ± 0.11 95 % CI of ±26.26 mmHg	0.86 (≥20 mmHg) 0.94 (≥40 mmHg)		
	Cardim et al. [19]	Assessment of *nICP_FVd* and comparison it with 3 other methods	40, TBI	Intraparenchymal	Correlation between ICP and nICP was *R* = 0.39* Bias of 7.34 ± 7.45 mmHg and 95 % CI of ± 14.62 mmHg R in time domain of -0.28 ± 0.69 For cases when ICP presented increases ≥ 7 mmHg (n = 8), the correlation between ICP and nICP was *R* = −0.32	0.70 (≥17 mmHg)		
	Cardim et al. [44]	Assess *nICP_FVd* as a nICP estimator and compares it with 3 other methods in NPH patients undertaking CSF infusion tests	53, NPH	Lumbar puncture	Baseline phase: Bias of 11.90 mmHg and 95 % CI of ±25.19 mmHg Plateau phase: Bias of 1.66 mmHg and 95 % CI of ±29.97 mmHg ΔICP vs ΔnICP correlation of −0.17 *R* considering changes of nICP and ICP in time domain was 0.35 ± 0.41			
*nICP_Edouard*	Edouard et al. [11]	Describe and assess a method for nCPP calculation based on FV and ABP under stable coditions and during CO_2_ reactivity test	20, TBI	Intraparenchymal	During normocapnia: nCPP and CPP were correlated (slope, 0.76; intercept, 10.9; 95 % CI, -3.5 to 25.4 mmHg) During hypercapnia: nCPP and CPP were correlated, but with increased discrepancy as reflected in confidence interval (slope, 0.55; intercept, 32.6; 95 % CI, 16.3 to 48.9 mmHg)			
	Brandi et al. [60]	Assessment of *nICP_Edouard*	45, TBI	Intraparenchymal	nICP: Bias of 6.8 mmHg and 95 % CI ± 19.7 mmHg nCPP: Bias of −6.8 mmHg and 95 % CI ± 45.2 mmHg			
*nICP_CrCP*	Varsos et al. [18]	Describe and assess a method for nCPP calculation based on FV (usnig the concept od CrCP) and ABP	280, TBI	Intraparenchymal	Correlation between nCPP and CPP was *R* = 0.85* Bias ± SD of 4.02 ± 6.01 mmHg nCPP estimation error was below 10 mmHg in 83.3 % of the cases Temporal analysis: Mean correlation in time domain was *R* = 0.73 (0.23–0.99) Bias of 3.45 mmHg (range: 4.69-9.03 mmHg) Mean SD of 5.52 mmHg (range: 1.52–10.76 mmHg) and 95 % CI of the SD of 1.89–5.01 mmHg	>0.8		
	Cardim et al. [19]	Assessment of nICP_CrCP and comparison with 3 other methods	40, TBI	Intraparenchymal	Correlation between ICP and nICP was *R* = 0.35* Bias of 4.44 ± 4.69 and 95 % CI of ±9.19 mmHg. *R* in time domain of 0.18 ± 0.56	0.70 (≥ 17 mmHg)		
	Cardim et al. [44]	Assess *nICP_CrCP* as a nICP estimator and compares it with 3 other methods in NPH patients undertaking CSF infusion tests	53, NPH	Lumbar puncture (CSF pressure)	Baseline phase of the test: Bias of 11.12 mmHg and 95 % CI of ±15.09 mmHg Plateau phase of the test: Bias of -2.53 mmHg and 95 % CI of ±17.80 mmHg ΔICP vs ΔnICP correlation of 0.21 *R* considering changes of nICP and ICP in time domain was 0.29 ± 0.24			

*AUC* area under the curve,* ABP* arterial blood pressure,* CI* confidence interval,* FV* cerebral blood flow velocity,* NPV* negative predictive value,* PPV* positive predictive value,* PE* prediction error, *R* correlation coefficient,* R* ^2^ coefficient of determination,* SD* standard deviation,* SAH* subarachnoid haemorrhage,* TBI* traumatic brain injury

* Correlation coefficient is significant at the 0.05 level

**Table 3:** nICP methods based on mathematical models

Method	Author	Study purpose	Sample size and disease	Invasive ICP monitoring	nICP method accuracy and correlation measures with ICP	Sensitivity (%)	Specificity (%)	AUC
*nICP_BB*	Schmidt et al. [20]	Describe and assess a method for nICP calculation based on FV and ABP using a black-box model	11, TBI	Epidural	MAD of 4.0 mmHg and SDE of 1.8 mmHg In this cohort a maximum 95 % CI of ±12.8 mmHg was found			
	Schmidt et al. [68]	This study aimed at predicting the time course of raised ICP during CSF infusion tests and its suitability for estimating the *R*_CSF_ using *nICP_BB*.	21, different types of hydrocephalus	Epidural	Analysis across all records: Correlation between *nR*_CSF_ and *R*_CSF_ was *R* = 0.73* MAD of 4.1 mmHg and SDE for *R*_CSF_ prediction of 2.2 mmHg × min/mL Analysis specific to different subtypes of hydrocephalus: Correlation between *nR*_CSF_ and *R*_CSF_ was *R* = 0.89* MAD of 2.7 mmHg and SDE for *R*_CSF_ prediction of 1.7 mmHg × min/mL			
	Schmidt et al. [69]	Assess *nICP_BB* during plateau waves of ICP	17, TBI (Plateau (A) waves observed in 7 patients)	Intraparenchymal	Correlation between nICP and ICP during ICP increase was *R* = 0.98* Analysis considering the baseline of plateau waves: MAD of 8.3 mmHg and SDE of 5.4 mmHg Analysis considering the top of plateau waves: MAD of 7.9 mmHg and SDE of 4.3 mmHg			
	Schmidt et al. [70]	This study aimed at investigating the ability of *nICP_BB* to adapt to the SCA, using Mx and PRx as parameters	145 (135 TBI, 10 Stroke)	Intraparenchymal/Intraventricular	Correlation between nMx and Mx was *R* = 0.90* Correlation between nPRx and PRx was *R* = 0.62* Median MAD of 6.0 mmHg Only TBI: MAD of 7.1 mmHg Only Stroke: MAD of 4.3 mmHg	For Mx: 97 For PRx: 61	For Mx: 92 For PRx: 67	
	Cardim et al. [19]	Assessment of nICP_BB and comparison with 3 other methods	40, TBI	Intraparenchymal	Correlation between nICP and ICP was *R* = 0.39* Bias of −0.50 ± 5.0 mmHg and 95 % CI of ±9.94 mmHg *R* in time domain was 0.48 ± 0.40			0.66 (≥17 mmHg)
	Cardim et al. [44]	Assess *nICP_BB* as a nICP estimator and compares it with 3 other methods in NPH patients undertaking CSF infusion tests	53, NPH	Lumbar puncture (CSF pressure)	Baseline phase of the test: Bias of 4.46 mmHg and 95 % CI of ±15.33 mmHg Plateau phase of the test: Bias of −7.35 mmHg and 95 % CI of ±19.21 mmHg ΔICP vs ΔnICP correlation of 0.30* *R* considering changes of nICP and ICP in time domain was 0.39 ± 0.43			
*nICP_Heldt*	Kashif et al. [24]	Describe and assess a method for nICP calculation based on FV and ABP	37 (45 TCD recordings in total, 30 bilateral), TBI	Intraparenchymal	Across all TCD records:	83	70	0.83 (≥20 mmHg)
					Correlation between nICP and ICP was *R* = 0.90 Bias of 1.6 and SDE of ±7.6 mmHg Inferred 95 % CI of ±14.9 mmHg Across bilateral TCD records: Correlation between nICP and ICP was *R* = 0.76 Bias 1.5 and SDE 5.9 mmHg. Inferred 95 % CI of ±11.6 mmHg			
					On a patient-record basis:	90	80	0.88 (≥ 20 mmHg)
					Correlation between nICP and ICP was *R* = 0.90 (*n* = 45 recordings)			
*Modified nICP_BB*	Xu et al.^26^	Describe and assess a method for nICP calculation based on FV and ABP, using an apprimorated model for *nICP_BB*	23, TBI, Hydrocephalus	Intraparenchymal/Intraventricular	MADs for non-linear models were <6.0 mmHg compared to 6.7 mmHg of the nICP_BB (linear) Inferred 95 % CIs for non-linear models were ≤±10.8 mmHg, compared to 10.6 mmHg of the linear model			
*Data mining*	Hu et al. [31]	Describe and assess a method for nICP calculation based on FV and ABP based on the concepts of data mining	9, TBI	Intraventricular	Median correlation between data mining nICP and ICP was *R* = 0.80			
	Kim et al. [32]	Describe and assess a method for nICP calculation based on FV and ABP based on the concepts of data mining	57, TBI	NM	Kernal Spetral Regression-based method presented a median Bias of 4.37 mmHg			
*Semisupervised learning*	Kim et al. [33]	Describe and assess a method for nICP calculation based on FV and ABP based on the concepts of semisupervised machine learning	90, TBI, SAH, NPH	Intraparenchymal/Intraventricular	Decision curve analysis showed that the semisupervised method is more accurate and clinically useful than the supervised or PI-based method			0.92

*ABP* arterial blood pressure, *AUC* area under the curve,* CI* confidence interval,* FV* cerebral blood flow velocity,* NM* not mentioned,* NPV* negative predictive value,* NPH* normal pressure hydrocephalus,* nR*_CSF_ nICP-derived R_CSF_, *MAD* mean absolute difference between ICP and nICP, * PPV* positive predictive value,* R* correlation coefficient,* R* ^2^ coefficient of determination,* R*_CSF_ resistance to cerebrospinal fluid (CSF) outflow,* SAH* subarachnoid haemorrhage,* SCA* state of cerebral autoregulation,* SDE* standard deviation of the error,* TBI* traumatic brain injury

* Correlation coefficient is significant at the 0.05 level. Inferred 95% CI was calculated as 1.96*SDE

## Results

### Methods Based on the Correlation Between ICP and PI (*nICP_PI*)

**Fig. 2:**
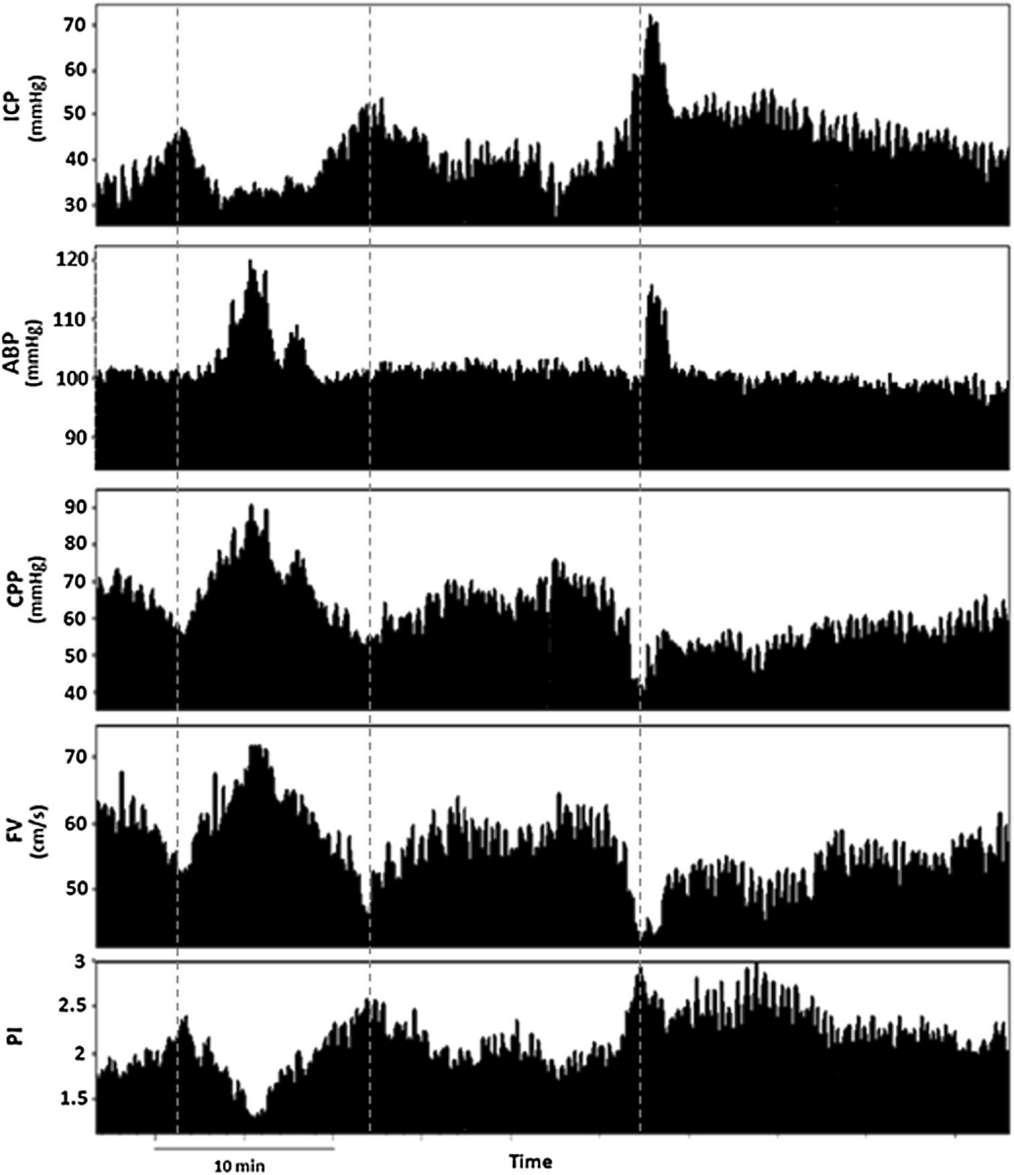
PI behavior during drop in CPP observed in a traumatic brain-injured patient (*source*: Brain Physics Laboratory TBI Database, University of Cambridge). *Dashed lines* represent periods when PI increased due to increase in ICP, independently of changes in ABP. *CPP* cereberal perfusion pressure, *PI* pulsatility index, *ICP* intracranial pressure, *ABP* arterial blood pressure, *TBI* traumatic brain injury

Pulsatility index describes quantitative and qualitative changes in the morphology of the TCD waveform resulting from cerebral perfusion pressure changes. It represents a relationship between the difference of systolic flow velocity (FVs) and FVd divided by mean flow velocity (FVm). All possible methods based on TCD-derived pulsatility index are based on observation that ICP and PI are positively correlated during increases of ICP. However, increase in PI is not specific for increase in ICP. In certain situations, such as a drop in CPP, PI presents an increasing trend, which can be related to increases in ICP or decreases in ABP (Fig. 2). The same behavior occurs during decrease in PaCO_2_ (partial pressure of carbon dioxide) or increase in pulsatility of ABP waveform. Mathematically, PI can be expressed as inversely proportional to mean CPP, directly proportional to pulse amplitude of arterial blood pressure and non-linearly proportional to the compliance of the arterial bed (Ca), cerebrovascular resistance (CVR), and heart rate (HR) [6].

Table 1 presents the papers which studied the relationship between PI and ICP. Accuracy of nICP estimation varies from ±5 to ±43.8 mmHg. The most favorable results are from Bellner et al. [7], in which the authors found a 95 % confidence interval for prediction of ±4.2 mmHg and strong correlation coefficient with ICP, *R* = 0.94 (*p* < 0.05). However, such results were never replicated by other authors.

### Methods Based on Estimation of CPP

The second approach for nICP monitoring was primarily intended for estimating the nCPP. However, non-invasive ICP can be calculated based on the assumption that nICP = ABP − nCPP. Four methods are described in the literature (Table 2).

#### nICP_Aaslid

Aaslid et al. [8] have determined CPP based on the amplitudes of the fundamental frequency components of FV (*F*) and of the arterial blood pressure (*A*):

$$ {\text{nCPP}} = {\text{FVm}}\,*\,A/F. $$

#### nICP_FVd

**Fig. 3:**
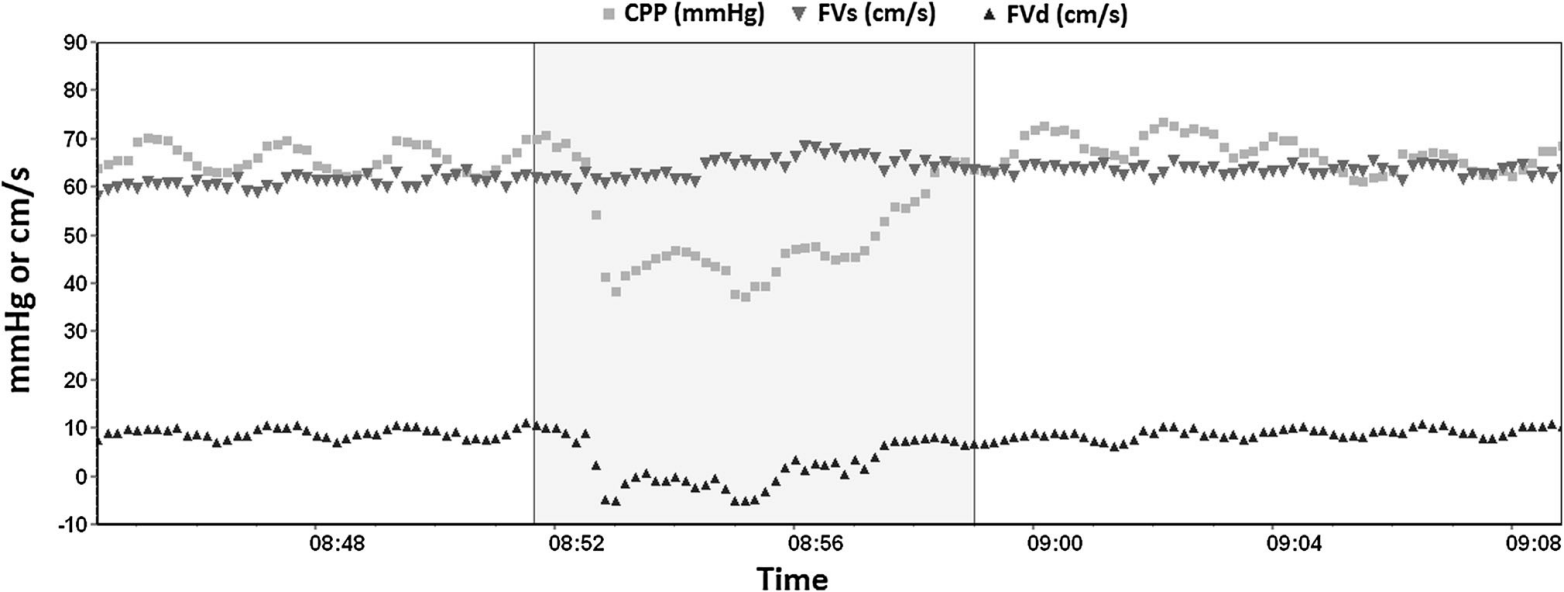
Systolic and diastolic flow velocities behavior during a drop of cerebral perfusion pressure during a plateau wave increase in ICP observed in a traumatic brain-injured patient (*source*: Brain Physics Laboratory TBI Database, University of Cambridge). FVd component in this case indicates inadequate cerebral perfusion. *CPP* cerebral perfusion pressure, *FVs* systolic flow velocity, *FVd* diastolic flow velocity, *ICP* intracranial pressure, *TBI* traumatic brain injury

Some studies have demonstrated that specific patterns of TCD waveform, such as a decrease in diastolic flow velocity, reflect impaired cerebral perfusion caused by a decrease in CPP [9, 10] (Fig. 3). Based on waveform analysis of FV [10], the proposed equation was

$$ {\text{nCPP}} = {\text{ABPm}} \times \frac{\text{FVd}}{\text{FVm}} + 14\,{\text{mmHg}} . $$

14 mmHg is a calibration (zeroing) parameter established for traumatic brain injury patients.

#### nCPP_Edouard

This method is based on the combination of the phasic values of both FV and ABP. The non-invasive CPP (nCPP) was calculated using the following formula [11]:

$$ {\text{nCPP}} = \left( {\frac{\text{FVm}}{{[{\text{FVm}} - {\text{FVd}}]}}} \right) \times ({\text{ABPm}} - {\text{ABPd}}) $$

where ABPm and ABPd are the mean and diastolic ABP, respectively.

#### nICP_CrCP

**Fig. 4:**
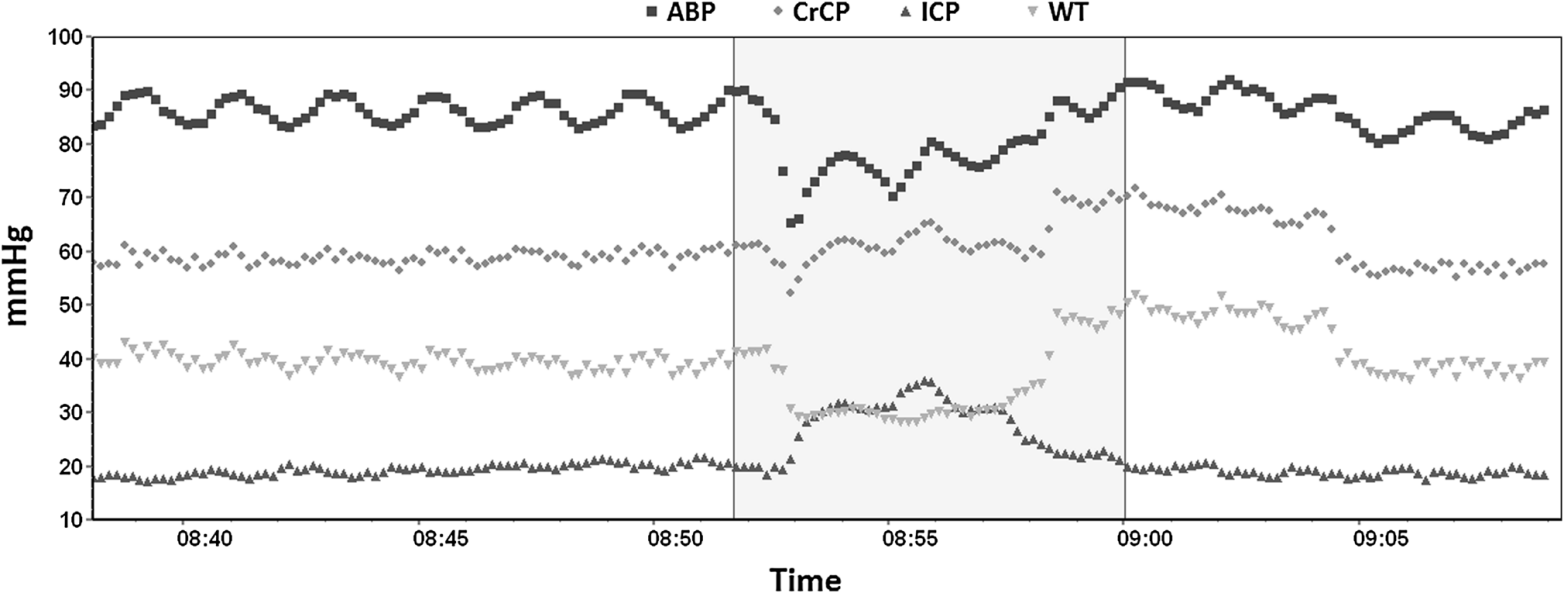
Representation of the CrCP interaction with ICP and WT in a situation of intracranial hypertension observed in a traumatic brain-injured patient (*source*: Brain Physics Laboratory TBI Database, University of Cambridge). During the increase of ICP, the CrCP also increases and WT decreases as an effect of preserved autoregulation. *ABP* arterial blood pressure, *CrCP* critical closing pressure, *ICP* intracranial pressure, *WT* wall tension, *TBI* traumatic brain injury

Critical closing pressure (CrCP) represents a threshold of ABP, below which the blood pressure in the brain microvasculature is inadequate to prevent the collapse and cessation of blood flow [12]. CrCP equals the sum of ICP and vascular wall tension (WT) [12, 13]: CrCP = ICP + WT. Given the association of this parameter with the vasomotor tone of small blood vessels (i.e., wall tension), CrCP may be able to provide information regarding the state of cerebral haemodynamics in several neurological conditions [12, 14–17] and then could reflect changes in CPP (Fig. 4). The equation for nCPP estimation based on CrCP is

$$ {\text{nCPP}} = {\text{ABP}} \times \left[ {0.734 - \frac{0.266}{{\sqrt {({\text{CVR}} \times {\text{Ca}} \times {\text{HR}} \times 2\pi )^{2} + 1} }}} \right] - 7.026 $$

$$ {\text{CVR}} = \frac{\text{CPP}}{\text{FV}} $$

$$ {\text{Ca}} = \frac{{{\text{CaBV}}\;{\text{Amp}}}}{{{\text{ABP}}\;{\text{Amp}}}}. $$

Constant coefficients (0.734, 0.266, 7.026) were derived from analysis of database of 232 TBI retrospective cases [18]. CaBV Amp represents the fundamental amplitude of the cerebral arterial blood volume. ABP Amp represents the fundamental amplitude of arterial blood pressure.

The accuracy for these methods was mainly reported for nCPP estimations and varied from ±12 to 48.9 mmHg. Variability for nICP in this case ranged from ±9.19 to ±59.60 mmHg. Out of the four methods, the best accuracy for prediction ICP was reported by Cardim et al. [19] elsewhere, using the *nICP_CrCP*, in which ICP could be predicted within a confidence interval of ±9.19 mmHg.

### Model-based nICP Methods

#### Black-Box Model for Estimation of ICP (*nICP_BB*)

**Fig. 5:**
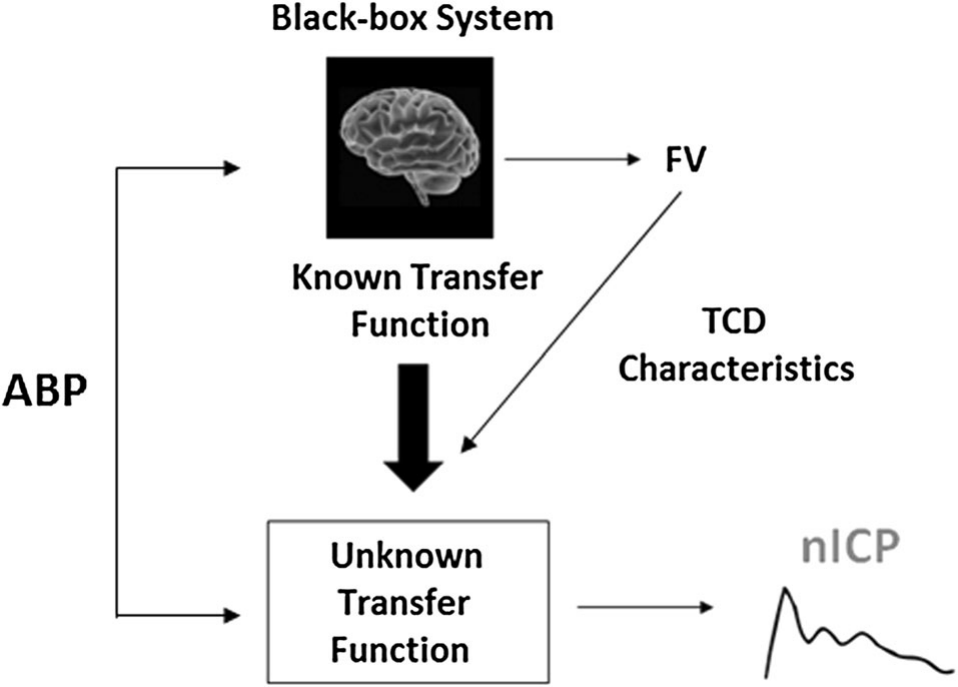
Schematic representation of the black-box model (Schmidt et al. [20]), for nICP estimation. A known transfer function (represented by a linear model) between ABP and FV, alongside modification (TCD) characteristics are used as means to dynamically define the rules for a transformation of ABP into nICP (unknown transfer function—a linear model between ABP and ICP). *ABP* arterial blood pressure, *FV* cerebral blood flow velocity, *TCD* transcranial Doppler, *nICP* non-invasive intracranial pressure

In this model, the intracranial compartment was considered a black-box (BB) system, with ICP being a system response to the incoming signal ABP [20]. The system response was described in terms of a transfer function between ABP and ICP [21, 22]. The transfer function was controlled by TCD and ABP derived parameters, the so-called TCD characteristics, which include ICP-related parameters and an ABP to TCD transfer function. The rules of this TCD-based linear control had been formerly determined using a multiple regression model between TCD characteristics and ABP-ICP transfer function on datasets of reference patients. The output data provide continuous full waveform of nICP (in mmHg) (Fig. 5).

Application of this model is summarized in Table 3.

#### Cerebrovascular Dynamics Model for Estimation of ICP (*nICP_Heldt* [23, 24])

This model-based nICP method focuses on the major intracranial compartments and their associated variables: brain tissue, cerebral vasculature, and cerebrospinal fluid (CSF). It continuously estimates and tracks ICP using measurements of peripheral ABP and FV in the middle cerebral artery (MCA). This physiological model of cerebrovascular dynamics is represented by a circuit analog and provides mathematical limits that relate the measured waveforms to ICP. Patient-specific ICP estimations are produced by an algorithm, with no calibration or training in specific populations needed. The dynamical model of CSF and cerebral blood circulation has been first published by Ursino and Lodi [25].

Accuracy of this method is summarized in Table 3.

#### Non-linear Regressions

##### Modified Black-Box Model

The previously described black-box model for ICP estimation [20] adopts a linear relationship among ABP, ICP, and FV. Xu et al. [26] assuming that the relationships among these three signals are more complex than linear models, and consequently not adequate to depict the relationship between ***f*** and ***w*** coefficients (relationship between ABP and ICP and that between ABP and FV, respectively), investigated the adoption of several nonlinear regression approaches. Considering that nonlinear regressions such as support vector machines (SVMs) [27], kernel spectral regression (KSR) [28] have been proved to be more powerful for the prediction problem than the linear ones [29, 30], the authors proposed to use these approaches to model the relationship between coefficients ***f*** and ***w***.

The ICP estimation showed that the mean ICP error by the nonlinear approaches can be reduced compared to the original approach (Table 3). Statistical tests also demonstrated that the ICP estimation error by the proposed nonlinear kernel approaches is statistically smaller than that obtained with *nICP_BB*.

##### Data Mining

Hu et al. [31] initially proposed an innovative data mining framework of nICP assessment. The proposed framework explores the rules of deriving ICP from ABP and FV that are captured implicitly by a signal database without using a mathematical model. The main strategy of the this framework is to provide a mapping function to quantify the uncertainty of an ICP estimate associated with each database entry, and to use this information to determine the best entry to build an ICP simulation model for an optimal ICP estimation. In comparison to Schmidt’s method (*nICP_BB*), for example, this model presented a smaller median normalized prediction error (bias), and a greater median correlation coefficient between estimated and measured normalized ICP (Table 3).

In another work of the same group, Kim et al. [32] aimed at adopting a new (linear and nonlinear) mapping functions into the previous data mining framework for nICP estimation to demonstrate that the performance of nICP assessment could be improved by utilizing proper mapping functions. Results are summarized in Table 3.

##### Semisupervised Learning

As previously seen, FV waveform analysis has been frequently applied for non-invasive ICP assessment. Kim et al. [33] introduced a non-invasive detection of intracranial hypertension method based on the TCD measurement of FV alone to demonstrate its performance both in the supervised and semisupervised learning settings (Table 3).

Out of these five model-based methods, the best accuracy was reported by Kashif and Heldt et al. [24], in which authors present a strong correlation between nICP and ICP of *R* = 0.90, sensitivity of 83 %, specificity of 70 %, with an AUC of 0.83 for detecting ICP ≥ 20 mmHg. In addition, a 95 % CI for prediction of ICP of ±14.9 mmHg (SDE of ±7.6 mmHg) was found for this method. Although other reviewed methods presented smaller 95 % CI, considering all measures of accuracy together, *nICP_Heldt* was the one showing the best performance.

## Discussion

Intracranial pressure and its management have been considered of fundamental importance in the treatment of neurocritical patients. ICP monitoring has been available since 1951, but it is important to realize that the monitor itself contributes little to outcome without proper interpretation and secondary analysis of the observed signal [34]. Instead, a positive outcome depends on how the data from the monitor are used and whether an effective treatments exists [34]. In a recent study, Chesnut et al., demonstrated that there was no difference in primary outcome in TBI patients who received ICP monitoring [35]. However, while this trial has internal validity, it has not been externally validated and did not test whether treatment of ICP per se makes a difference, but rather compared two management protocols (patients with or without ICP monitoring) [34]. Moreover, ICP should not be considered solely as a “number,” as waveform analysis of this parameter is also important [36]. For instance, ICP waveform analysis can provide information on the state of cerebrovascular reactivity (PRx index), compensatory reserve (RAP index) and can be used to estimate individualized optimal cerebral perfusion pressure levels [37, 38].

Despite eventual complications that might raise from invasive monitoring, direct methods still remain as the gold standards [2]. When direct ICP monitoring is contraindicated, a reliable non-invasive method would be helpful, at least in the early stages of treatment, when it could act as a screening tool. Such scenario would be beneficial to a wide range of neurological conditions in which ICP monitoring is not usually applied or is a neglected parameter, such as cerebral malaria [39, 40], status epilepticus, mild or moderate TBI, brain tumors. For an example, TCD has been demonstrated to accurately screen patients with mild or moderate TBI at risk of secondary neurological deterioration [41].

The advent of Transcranial Doppler Ultrasonography allowed the development of several methods dedicated to assess the cerebrovascular circulation and dynamics, particularly non-invasive assessment of ICP and CPP. Moreover, subject to good fixing of ultrasound probes, it allows monitoring of these parameters as they may change in time. TCD comprises most of the features a nICP method should contemplate: relatively low cost, risk-free, easily available, portable, high temporal resolution, repeatable and suitable for emergency and ambulatory settings. Nevertheless, as most of the non-invasive techniques, TCD also presents some intrinsic disadvantages which can negatively influence its accuracy. They are mainly represented by signal transmission attenuation through the cranial bones, linearity and stability of the signal in time. Furthermore, TCD measurements may be especially difficult in a certain percentage of the population (up to 8 %) which does not present an adequate acoustic window for artery insonation [42]. On the other hand, accuracy may not be the primary performance measure in every clinical situation, and such downsides may be compensated for by the ability of the method to track changes and trends of ICP over time, rather than its absolute value.

**Fig. 6:**
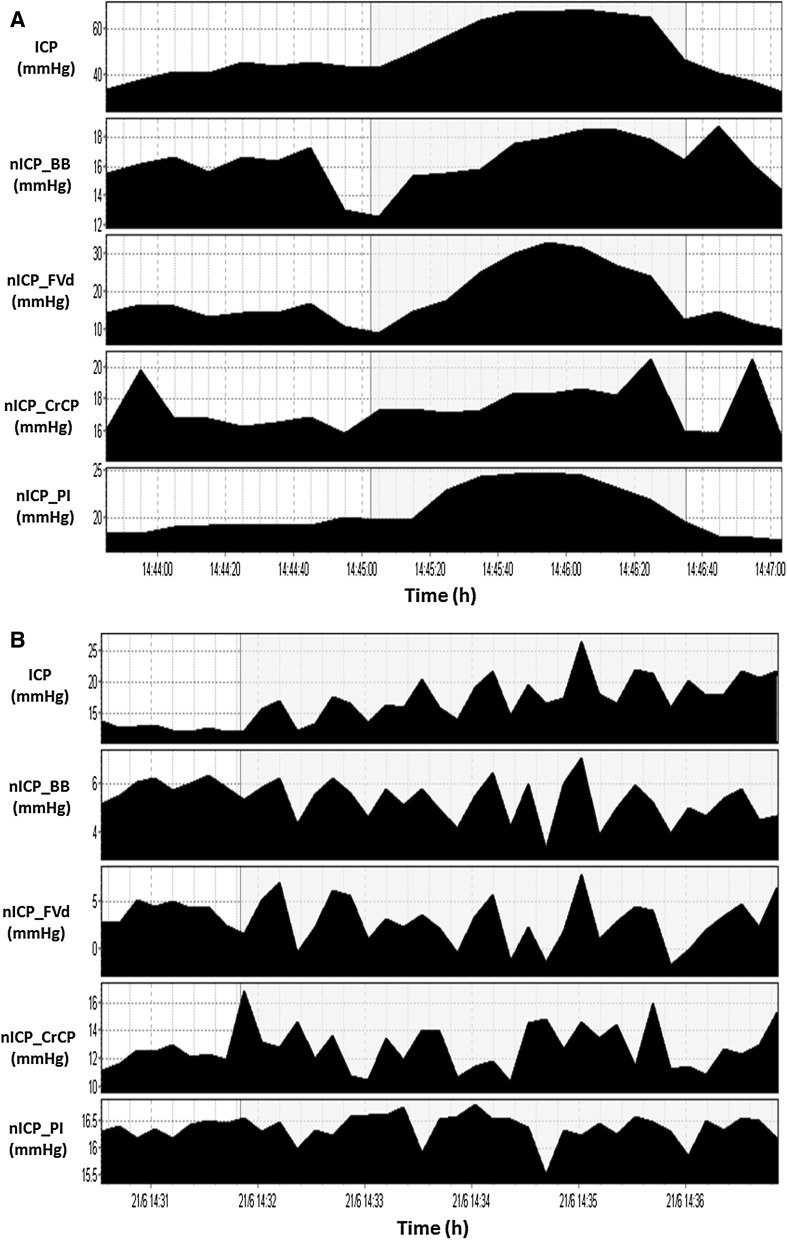
Example of vasogenic waves during CSF infusion test (Cardim et al. [44]). *Shadowed areas* in (**a**) and (**b**) represent ICP waves of vasogenic origin. It is possible to observe that at least for trends in time, there were good correspondence between ICP and nICP methods; *nICP_BB* non-invasive ICP method based on mathematical black-box model [6]; *nICP_FVd* non-invasive ICP method based on FVd [10]; *nICP_CrCP* non-invasive ICP method based on CrCP [18]; *nICP_PI* non-invasive ICP method based on PI

In addition, a possible disadvantage about TCD is related to its principle of working. It is known that this technique is limited to detecting changes in the arterial bed of vasogenic origin [43] (i.e., changes of arterial blood volume). Considering that ICP consists of several components (i.e., inflow and volume of arterial blood, venous blood outflow, CSF circulation and brain parenchyma volume), it is expected that a TCD-based method would present lower accuracy if changes in ICP were caused, for example, by derangements in the CSF circulation or by increase in parenchyma volume, rather than if they were purely of vasogenic origin. This is mainly because changes in the CSF and brain parenchyma compartments would not be promptly transmitted to the arterial bed as of those of vasogenic origin. Such characteristic can be exemplified by the fact that in certain situations where changes of ICP related to vasogenic fluctuations (plateau waves, B waves) overlaps the changes of ICP related to CSF circulation (for instance during CSF infusion test), there is a strong correlation in time domain between real and TCD-estimated ICP (as seen in Fig. 6, obtained from Cardim et al. [44]), even showing reliable replications of vasogenic waves patterns.

According to the revision of the presented methods, the measures of accuracy for each method varied substantially within and among nICP categories as observed in Tables 1, 2 and 3. For the approaches based on mathematical models, for instance, the most frequent measures were the ‘Bias’ and ‘SDE’ (standard deviation of the error [bias]), and MAD (mean absolute difference).

Over measures of accuracy, a standard statistical assessment would be interesting for works on non-invasive intracranial pressure methods. For instance, at least for a clinical point of view, an assessment should contain the following statistical indicators: (I) correlation between nICP and measured ICP considering mean values of ICP and changes of ICP in time domain; (II) Bias and 95 % CI for prediction of ICP; (III) ROC analyses including the nICP method prediction ability at a certain threshold (usually around 20 mmHg for intracranial hypertension), sensitivity and specificity. Altogether, these parameters should provide the clinician a comprehensive picture of the qualities and downsides of a method.

Another aspect that might confuse interpretation and comparisons is how the nICP averages were obtained, i.e., every work present with different average calculation windows. The number of samples should also be considered when comparing such results. Moreover, information about sensitivity and specificity, confidence intervals for prediction or any of the above mentioned parameters is not systematically available in the majority of the reviewed papers. Some of the studies also include a small number of patients, making a quantitative comparison unfeasible. This variability illustrates the importance and necessity of studies applying the same number of samples, calculation methods and measures of accuracy in order to compare different nICP methods consistently.

The several degrees of approximation for ICP monitoring (i.e., epidural, subdural, intraparenchymal and intraventricular) could also contribute to a misleading interpretation when validating nICP methods against different invasive techniques. This is due to the presence of multiple intracranial compartments of variable deformability and ability to transmit pressure. Thus, ICP needs to be considered as an anisotropic parameter rather than a global isotropic pressure equally distributed in all intracranial compartments. Over this concept, each invasive method would then be specific to measuring compartmental pressures according to where they are located in the intracranial system. As proofs of this concept, simultaneous measurement of ICP by intraparenchymal and intraventricular probes showed a bias of −1.2 and a 95 % CI of ±6.8 mmHg (SDE of ±3.4 mmHg) [45]. In another study, simultaneous measurements of ICP using intraparenchymal and epidural probes presented a bias of 4.3 mmHg, with 95 % CI of ±17 mmHg (SDE of ±8.5 mmHg) [46]. Under these circumstances, the characteristics of invasive ICP monitoring should also be considered in the standard nICP assessment.

Nevertheless, qualitative-wise, TCD-based nICP methods generally presented a positive degree of agreement and acceptable correlations with measured ICP (or with CPP for nCPP-based methods), with exceptions for PI-based methods. For this category, even though most of the studies indicated a direct, or at least indirect, correlation between PI and ICP, there is a divergence whether PI can predict ICP reliably, with some studies showing rather weak or even inexistent correlations between these two parameters [6, 19, 47–51]. Such a controversy might originate from the different conditions in which PI can increase independently of increases in ICP.

Considering the 95 % CI for the presented methods, there is a wide variability for the different method categories (from ±4.2 to ±59.60 mmHg). The authors’ personal experience on working with TCD-based methods suggests there is an overall intrinsic confidence interval of around ±12 mmHg, which still needs to be extensively validated in different patient populations and clinical conditions. Provided that the clinically relevant range of ICP is about 10 or 20 mmHg, TCD-based methods at the current state of development are not able to predict mean values of ICP with great confidence. However, the cerebral circulation dynamics can be observed with such methods as nICP changes in time domain, and tracked in real-time in the clinical setting (as observed in Fig. 6, for instance). This is one of the advantages of Transcranial Doppler Ultrasonography and may become particularly useful as a primary assessment tool in centres where ICP measurements are not routinely applied, or in patients in whom ICP monitoring is unavailable or may not be clearly indicated.

In conclusion, although Transcranial Doppler Ultrasonography consists of a technique with various possibilities for nICP estimation, there is still a necessity of studies to systematically compare them in different clinical conditions, in order to determine which approach offers the best reliability to monitor ICP dynamics non-invasively.
